# Ferrofluid droplet generation on a zero-thickness nozzle by a magnetic field using a wedge-shaped functional surface

**DOI:** 10.1371/journal.pone.0321099

**Published:** 2025-05-23

**Authors:** Amirhossein Favakeh, Mohamad Ali Bijarchi, Mahbod Mohammadrashidi, Mohammad Yaghoobi, Mohammad Behshad Shafii

**Affiliations:** 1 Center of Excellence in Energy Conversion (CEEC), Department of Mechanical Engineering, Sharif University of Technology, Tehran, Iran; 2 Sharif Energy, Water, and Environment Institute (SEWEI), Tehran, Iran; National Research Centre, EGYPT

## Abstract

Digital microfluidics for ferrofluids enables the manipulation of discrete droplets on open surfaces and has garnered significant interest as an alternative to traditional continuous-flow microfluidic systems. However, droplet generation within digital microfluidics remain underdeveloped. This study introduces a novel method for droplet generation using a wedge-shaped surface with hydrophilic-hydrophobic patterning, which functions as a two-dimensional flat nozzle. We first demonstrated the concept by investigating gravity-driven water droplet generation on a sloping surface, revealing that smaller droplets form at higher tilting angles, while droplet size remains constant with increasing flow rate. Frequency of droplet formation decreases by 60% with decreasing the tilting angle from 90° to 30°. The proposed method results in significant improvement in frequency (10 Hz) compared to nozzle-based droplet generation (1–5 Hz). We then extend this approach to ferrofluid droplets under an external magnetic field, observing five distinct steps in the formation process. Additionally, a scale analysis of both water and ferrofluid droplet generation provides a deeper theoretical understanding of the governing forces, showing a strong correlation between non-dimensional droplet diameter and the Bond number, following a -1/3 power law (R^2^ > 0.95). The derived empirical factor offers precise droplet diameter predictions, with an average error of 3.9%. Finally, inspired by cactus structures, we demonstrate parallelization of the flat nozzles, highlighting the potential for high-throughput droplet generation in digital microfluidic applications.

## Introduction

Digital microfluidics serves as a basis for droplet-based microfluidics in the range of nano to microliter scale and deals with manipulating droplets on open surfaces without any confinement [1]. Proteomics [2], immunoassay [3], clinical diagnostics [4], and cell culture can be mentioned as some of the applications of digital microfluidics. Unlike continuous-flow droplet-based microfluidics, digital microfluidics provides the controllability of individual droplets on open surfaces, which can be utilized as a discrete reaction chamber. An external stimulus is required to actuate the droplets, amongst which Electrowetting-on-Dielectric (EWOD) [5,6], Surface Acoustic Waves (SAW) [7], and magnetic schemes [8–11] could be mentioned. Magnetic actuation presents unique benefits such as scalable handled liquid volume, contactless control of the sample, the flexible and reprogrammable fluidic path, multi-functionality of a single digital microfluidic design, and the possibility of employing a wide range of liquid properties [12].

The generation of droplets is a critical part of some microfluidic assays [13]. An effective droplet generation method should allow high-throughput production and precise control over droplet size. Droplet generation techniques are generally categorized into active and passive methods [14]. In passive techniques, no external stimulus is applied, so the characteristics of the droplet formation depend solely on the design and geometry of the system, surface wettability, flow rates, and fluid properties. Pressure-driven droplet generation in microfluidic T-junctions [15,16] and step-emulsification microchannels [17] are the most common passive techniques. The main drawback of the passive techniques is the lack of control over the size and formation rate of the droplets. Other passive techniques, such as droplet microarrays, have more control over the droplet size, but the throughput of these systems depends on microarray density on the surface, and the droplets form in specified locations [18]. In contrast, active techniques use an external energy source—such as electrical [19,20], mechanical [21,22], and magnetic energies [23]—to initiate droplet formation and manipulation and offer greater control over the process [24].

Ferrofluid is a colloidal suspension of magnetic particles with less than 10 nm diameters coated with a surfactant in a carrier liquid [25–27]. Due to its responsiveness to external magnetic fields, ferrofluid has attracted significant interest in microfluidics applications [28,29]. In continuous-flow microfluidics, ferrofluid has been widely utilized in operations such as micromixers [30] and micropumps [31]. In digital microfluidics, Bijarchi et al. experimentally investigated the possibility of employing an adjustable alternating magnetic field to manipulate ferrofluid droplets on surfaces [32–34]. Ferrofluid droplets can also be coated by a layer of hydrophobic particles [35,36,37] to form “ferrofluid marbles,” allowing them to easily roll on surfaces with minimum friction and without wetting the surface.

An external magnetic field can serve as an energy source in droplet generation, enabling remote and precise control over the droplet generation process. Kahkeshani and Dicarlo examined the ferrofluid droplet generation in a step emulsification device, finding that the droplet size mainly depends on the step geometry [38]. Yet, these passive methods lack the required control for precision applications. Bijarchi et al. also investigated the generation of ferrofluid droplets in a T-junction under a pulse-width modulated magnetic field [39]. They also numerically [40] and experimentally [41–44] studied the ferrofluid droplet generation in a nozzle. An external magnetic field could also manipulate non-magnetic droplets on a ferrofluid-infused substrate [45]. However, the soft lithography techniques for fabricating enclosed microfluidic channels [46] for these designs make these platforms costly and less accessible. In contrast, surface patterning in digital microfluidics is more cost-effective and easier to fabricate.

Wedged-shaped functional surfaces have been widely used in digital microfluidics for fluid transportation [47]. Due to the unbalanced capillary forces caused by the triangular shape of the wedges, a droplet spontaneously moves when placed on a wedged-shaped surface [48,49]. Liu et al. investigated the directional motion of water droplets on a wedged-shaped surface, where the wedged region is hydrophilic, and the outer area is hydrophobic [50]. They experimentally and theoretically revealed that the transport behavior of droplets mainly depends on the wedge geometry, contact angle of the hydrophilic region, and droplet volume. Ody et al. studied the controlled motion of ferrofluid droplets on a wedged-shaped surface under external magnetic fields. They found that the droplet stops when a magnetic field is applied perpendicular to the surface tension gradient [51]. These studies highlight the importance of digital microfluidics in droplet manipulation and transport [52]; however, there remains a gap in the applicability and feasibility of these systems for efficient droplet generation. Ferrofluid droplet generation has been extensively studied numerically and experimentally through microfluidic chips and nozzles [53,54], which make the downstream process impossible or costly. Further, there still exists a gap in the possibility that we can integrate ferrofluid droplet formation systems with manipulation designs [55], which would be significantly easier with open-surface microfluidics.

This work introduces a new technique for droplet generation in open-surface microfluidics using wedge-shaped functional surfaces as two-dimensional flat nozzles to generate droplets that can be collected and manipulated downstream within integrated systems. This approach holds fluid on a hydrophilic, wedge-shaped surface, and droplets detach when an external driving force is applied. First, we demonstrated this concept by generating water droplets under gravitational force via surface tilting. We then extended the method to generate ferrofluid droplets, studying the effects of wedge geometry and magnetic field strength. To the best of the authors knowledge, this is the first study that introduces a method of ferrofluid droplet generation using magnetic field in open channel microfluidics. Actually, we do not claim that the proposed method in this paper can generate faster, smaller, or monodispersed droplets relative to well-known procedures like EWOD. It is the first idea to generate droplets using magnetic field on an open surface which can be used for droplet manipulation in the next step. Through scale analysis, we derived correlations that aid future designers in optimizing this technique. We further demonstrated parallel droplet generation on multiple flat nozzles inspired by cactus morphology. This method addresses a key gap in digital microfluidics by providing a cost-effective alternative to confined microchannel designs, relying only on hydrophobic-hydrophilic patterning [56] on flat surfaces, thus significantly reducing fabrication complexity and costs.

## Materials and methods

The camera used in the study was the Nikon-1 J4. We recorded the droplet formation process with a frame rate of 1200 fps. A syringe pump (Fnm, SP1000HPM) was used for the injection of either water or ferrofluid.

A cubic magnet with a length and width of 5 cm and a height of 2.5 cm has been used in the experiments. The magnetic flux density in the horizontal (x) and vertical (z) directions against the distance from the magnet are measured by an MG3002-LUTRON Gaussmeter with an accuracy of 0.01 mT and are shown in Fig 5.

**Fig 1:**
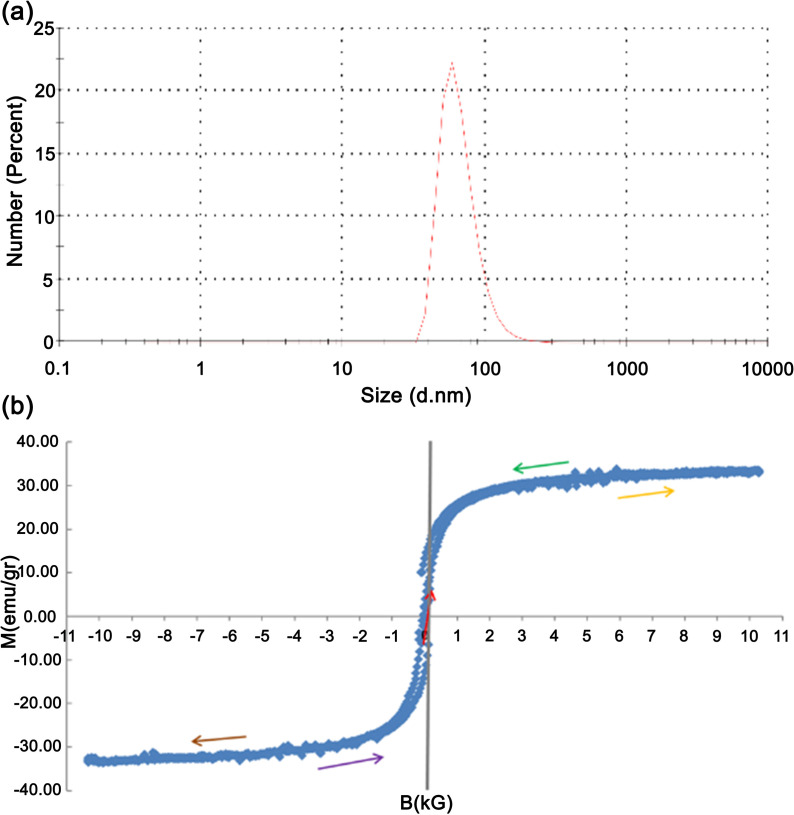
(a) The diameter distribution of magnetic nanoparticles and (b) the magnetization curve of the ferrofluid.

**Fig 2:**
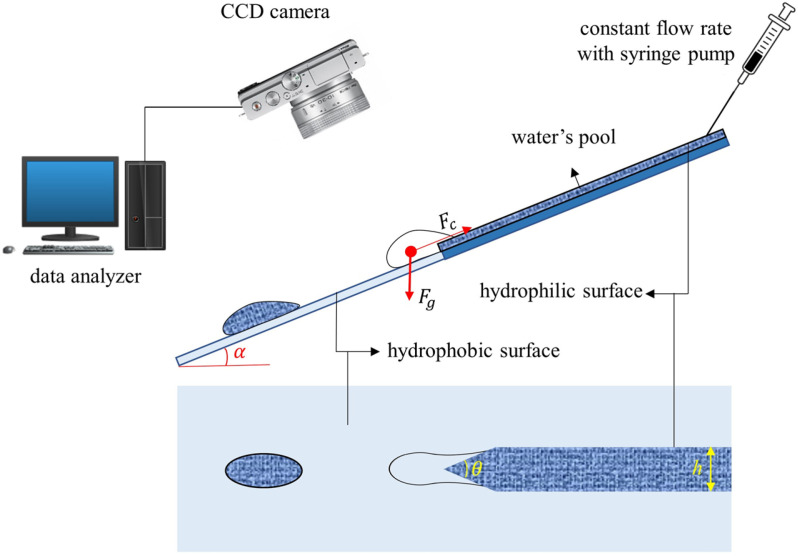
The proposed system for the formation of water droplets under gravity.

**Fig 3:**
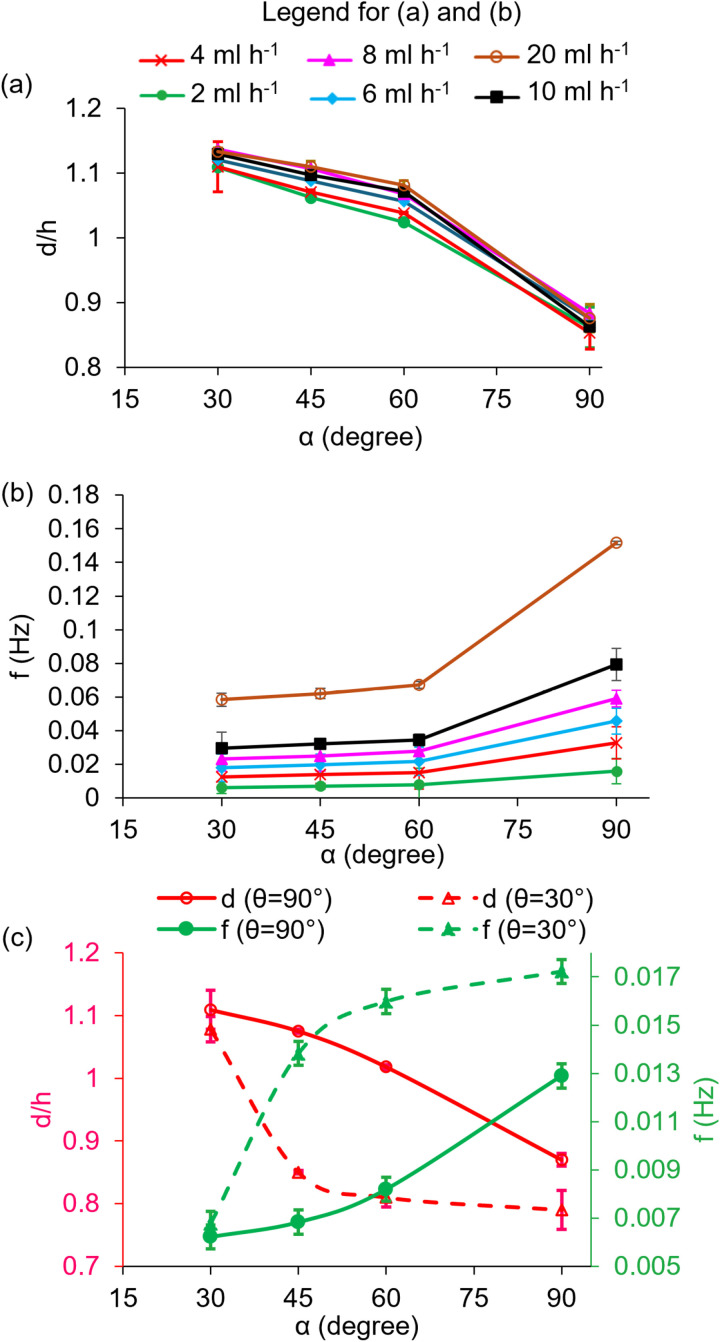
Variations of (a) non-dimensional diameter and (b) the frequency of droplet generation in terms of the tilting angle for different water flow rates. The vertex angle is 90°. (c) non-dimensional diameter and frequency of droplet generation against the tilting angle at vertex angles of 30° and 90° at a constant flow rate of 2 ml h^**-1**^.

**Fig 4:**
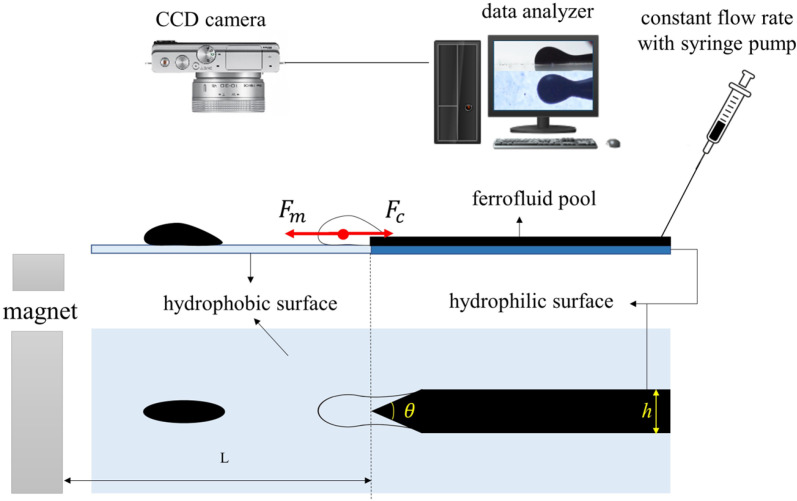
Schematic of the experimental setup for the generation of ferrofluid droplets using a magnet.

The ferrofluid used in this study is a water-based ferrofluid synthesized with the co-precipitation method. To prepare the ferrofluid, first, the deoxygenated distilled water is poured into a chamber, and then argon gas is injected into the chamber throughout the process to remove the remaining oxygen gas so that the iron (III) ions do not convert to iron (II) in the presence of oxygen. One of the signs of the conversion of iron ions III to II is the change in color of the solution from black to brown. 10.128 grams of 6-aqueous ferric chloride salt (FeCl_3_.6H_2_O) and 3.976 grams of 4-aqueous ferrous chloride salt (FeCl_2_.4H_2_O) are added to the distilled water.

These surfactants create biocompatibility in droplets that can carry a biological component for medicinal purposes. Finally, distilled water is added until the volumetric concentration of nanoparticles reaches the desired amount (1.3% in this study) and then placed in the sonicator for ten minutes. The average diameter of surfactant-coated nanoparticles is 66.97 nm (Fig 1a). The magnetization curve of the ferrofluid is shown in Fig 1. (Princeton Applied Research, Vibrating Sample Magnetometer, Model No. 155, Magnet: Varian, V-7300 Series 12” Electromagnet)

A volumetric nanoparticle concentration of 1.3% water-based ferrofluid has been used in all experiments. The viscosity and density of ferrofluid are 4.66 ± 0.01 mPa.s and 1097.0 ± 0.1 kg/ m^3^, respectively, which have been measured by viscometer and density meter. The surface tension of ferrofluid has been measured at 45.0 ± 0.1 m N m^-1^ by a tensiometer device (KRUSS GmbH Germany, K6).

The materials for creating a surface pattern cost around $10, while a soft lithography device can cost up to $100, making them less accessible in certain situations

The contact angle of ferrofluid droplets on the hydrophilic and hydrophobic surfaces was measured by image processing. According to Table 1, contact angles were obtained as 93 and 13 degrees for the hydrophobic and hydrophilic surfaces, respectively.

**Table 1:** Comparison of measured contact angle of ferrofluid droplets on hydrophilic and hydrophobic surfaces.

Surface type	Shape of droplets on the surface	Contact angle
hydrophobic	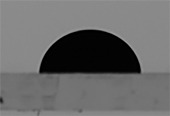	93˚ ± 3˚
hydrophilic	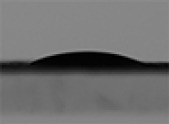	13˚ ± 4˚

## Results and discussion

### Proof of the proposed concept with sloping surface and fluid water

Herein, the proposed idea is simply demonstrated using water. In this technique, two factors are needed for droplet formation:

- A functional wedged surface with a hydrophilic-hydrophobic design (flat nozzle): As shown in Fig 2, the patterned part represents the hydrophilic surface, while the outer part is hydrophobic. For this purpose, a hydrophilic glass slide is used. To make the hydrophobic part, a piece of adhesive tape with the desired shape is attached to the hydrophilic part of the surface as a cover mask, and then the rest of the surface is coated by silicon oxide particles; therefore, the patterned part retains its hydrophilicity whereas the surrounding area becomes hydrophobic. The hydrophilic part is a rectangle with a width of 5 mm, which ends in a triangle with a vertex angle of *θ*.
- Driving force for droplet generation: By tilting the glass slide with the slope of α, a component of the gravitational force (weight) is exerted in the direction of the surface, which can cause the droplet to detach from the liquid reservoir held by the surface tension exerted by hydrophilic part. By changing the tilting angle (*α*), the gravitational force is adjusted by ρgsinα, where ρ and g are, respectively, fluid density (kg m^-3^) and gravitational acceleration (m s^-2^), is adjusted.

Water is injected into the hydrophilic surface with a constant flow rate (Q) of 2–20 ml h^-1^. The volume of the detached droplet is obtained by measuring the frequency of droplet formation using the law of conservation of mass $(\frac{V_{d}}{f}=Q$). Subsequently, the effective diameter of droplets is obtained by Equation (1).


$$d={\left(\frac{6}{\pi }\frac{Q}{f}\right)}^{\frac{1}{3}}$$ (1)

In the experiments, the tilting angle was adjusted to 30, 45, 60, and 90 degrees. The droplet formation was also examined with vertex angles of 30° and 90°. The diameter of the droplet is expressed in the dimensionless form (d/h), where h is the width of the hydrophilic part.

Two forces of gravity ($F_{g}$) and capillary ($F_{c}$) are influential in droplet formation. Fig 3 illustrates the droplet diameter and frequency of the droplet generation in terms of the tilting angle for different water flow rates. By increasing the tilting angle (α), the component of the gravitational force parallel to the surface increases. Consequently, the gravitational driving force overcomes the opposing capillary force at smaller volumes of droplets, resulting in a faster detachment of droplets with a smaller volume; therefore, for all flow rates, by increasing the tilting angle, the diameter of droplets decreases, and the frequency of droplet formation increases. The gravitational Bond number is defined as $Bo=\frac{\rho gd^{2}\sin\alpha }{\gamma }$, where γ as the surface tension. The interesting observation is that by changing the flow rate, none of the two forces involved in droplet formation change, and thus, the droplet diameter remains almost constant (Fig 3a). The increase in the flow rate only affects the frequency of droplet formation and causes a faster detachment of droplets (Fig 3b).

Further, Fig 3c shows that the diameter of the droplets at the vertex angle of 30° is less than that of 90°, while the droplet generation frequency is higher. In fact, by decreasing the vertex angle at a constant tilting angle, the applied gravitational force remains unchanged; however, the tip of the nozzle is sharper and narrower at the vertex angle of 30° than 90°, and thus, the exerted capillary force on the droplet is smaller at the vertex angle of 30°, resulting in faster-forming droplets with smaller volume.

### Ferrofluid droplet generation

To increase the controllability of the system and to allow the active generation of droplets on non-sloping surfaces so that it can be used in the applications mentioned in the digital microfluidic system, water-based ferrofluid is used instead of water, and a permanent magnet is utilized as the external stimulus. The proposed system can address the challenges of droplet generation in digital microfluidic systems with precise control over the droplet size and the droplet generation rate.

Fig 4 shows a schematic of the experimental setup for generating ferrofluid droplets using a magnet.. Similar to the previous section, two factors of a hydrophilic-hydrophobic patterned surface and an external driving force are required to generate droplets. The same hydrophilic-hydrophilic surface is used, but ferrofluid is used instead of water, and the magnetic force is used instead of gravity, and there is no need to slope the surface; therefore, magnetic (*F*_*m*_) and capillary (*F*_*c*_) forces are the most dominant forces in the process of ferrofluid droplet generation.

In this part of the experiment, ferrofluid with a constant flow rate in the range of 2–20 ml h^-‍1^ is injected from the right side of the hydrophilic surface. The droplet generation process is recorded after reaching a steady state condition. Contrary to the previous part, where the tilting angle of the surface was adjusted to alter the driving force, in this part, the driving magnetic force is tuned by changing the distance of the magnet from the tip of the hydrophilic part.

**Fig 5:**
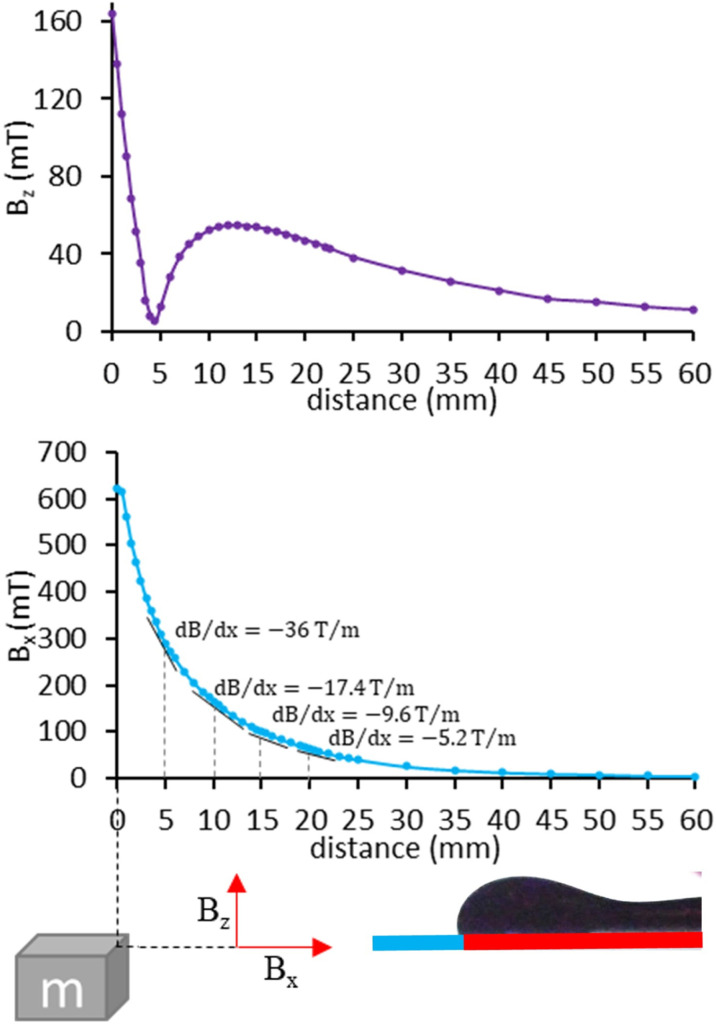
Magnetic flux density in the x and z directions against the distance from the magnet.

The magnetic flux density in the x-direction in Fig 5 is dominant over the z-direction, and thus, the magnetic driving force is almost horizontal. The small component of the magnetic flux density in the z-direction leads to the exertion of a negligible force perpendicular to the surface, which causes the deformation of the droplet.

**Fig 6:**
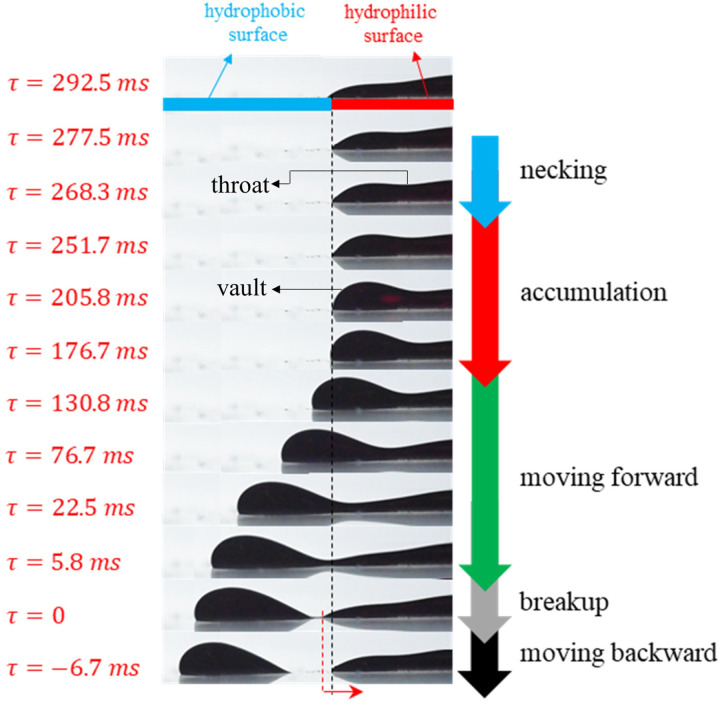
Ferrofluid droplet generation on a functional wedged surface atθ = 20°, Q = 5 ml h^-‍1^ and L = 10 mm. τ = t_breakup_ -t represents the remaining time until the droplet formation.

Fig 6 and Movie S1 show the process of generating ferrofluid droplets on a wedged surface with a vertex angle of 20°, a flow rate of 5 ml h^-1^, and a magnet distance of 10 mm from the vertex of the hydrophilic part.

It is observed that by injecting ferrofluid on the hydrophilic surface, the fluid accumulates on the hydrophilic surface and does not exceed the hydrophobic part of the surface. By injecting more volume of ferrofluid, a throat is formed in the ferrofluid layer in the hydrophilic part. Over time, ferrofluid accumulates at the edge of the hydrophilic part and forms a vault near the hydrophilic-hydrophobic boundary, but it is not yet able to overcome the capillary force to move onto the hydrophobic surface, which leads to the further accumulation of ferrofluid. By increasing the volume of the accumulated ferrofluid, the magnetic force overcomes the capillary force. Consequently, the ferrofluid interface gets pulled toward the hydrophobic part while the thickness of the throat decreases (moving forward). Eventually, a piece of ferrofluid is detached on the hydrophobic surface slightly ahead of the hydrophilic-hydrophobic boundary, generating a droplet (breakup). The remnant ferrofluid on the hydrophobic surface is then moved backward by the surface tension force (moving backward).

**Fig 7:**
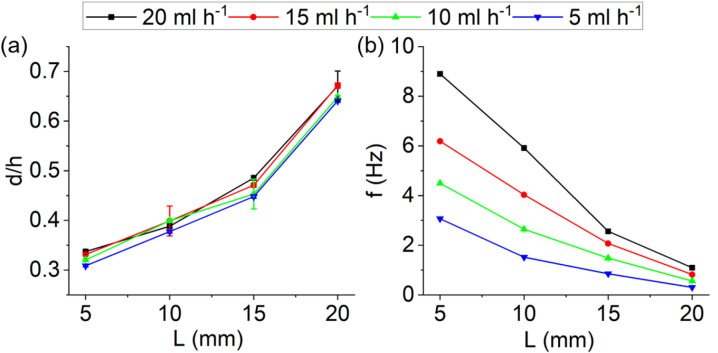
(a) Dimensionless diameter and (b) droplet generation frequency versus the magnet distance from the vertex of the hydrophilic surface for different ferrofluid injection rates and the triangle vertex angle of 10°. Each measurement is an average of at least five droplets. The standard deviation is significantly small and is not visible.

The effect of the magnet distance from the vertex of the hydrophilic surface and the ferrofluid injection rate are shown in Fig 7. As the distance of the magnet from the vertex of the hydrophilic surface increases, the magnetic flux density decreases, and consequently, the magnetic driving force decreases. Hence, the droplet is formed with more volume and lower frequency so that the magnetic force overcomes the opposing capillary force. It is also observed that as the flow rate changes, the diameter of the ferrofluid droplet remains almost constant because none of the magnetic and capillary forces change. The effect of the flow rate change is only observed on the droplet generation frequency, whereby by increasing the flow rate, the droplet will be formed with a higher frequency.

At the highest flow rate, the frequency of ferrofluid droplet generation simply exceeds the nozzle-based droplet generation by two times which increases the throughput of the proposed method in comparison to state-of-the-art systems [32,39,40] as such in these references the frequency of droplet generation is at most around 1–5 (Hz). However, the current method gives the possibility of improving the throughput frequency to 10 (Hz); significantly more than state-of-the-art. But there is no limit to increasing the flow rate to further increase the throughput.

**Fig 8:**
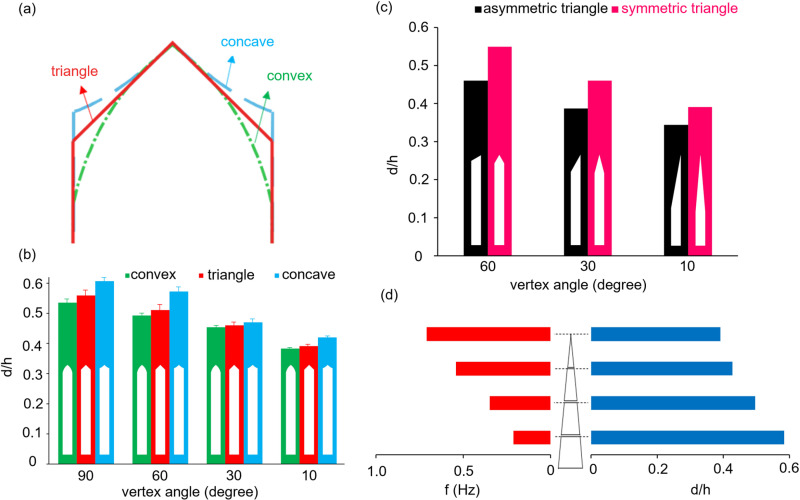
(a) Convex, triangular, and concave geometry at a constant vertex angle. (b) Comparison of the effect of the hydrophilic part geometry, including convex, triangle, and concave, on the diameter of the droplet formed at different vertex angles for constant values of Q = 10 ml h^-‍1^ and L = 10 mm, (c) Comparison of the effect of the symmetric and asymmetric triangles on the diameter of the droplet formed at different vertex angles for constant values of Q = 10 ml h^-‍1^ and L = 10 mm. (d) Variations of dimensionless diameter and droplet generation frequency for trapezoidal geometry with top sides of 1.5, 2.3, and 3.5 mm and its comparison with a triangle with a vertex angle of 10° for constant values of Q = 10 ml h^-‍1^ and L = 10 mm.

The effect of changing the geometry of the hydrophilic part is investigated in three cases. In the first case, as shown in Fig 8a, the concave, triangular, and convex geometries are compared at different vertex angles. Fig 8a shows that at each vertex angle, the concave and convex geometries are designed to be tangent to the vertex angle of the triangle. As shown in Fig 8b, the diameter of the droplet decreases in all three cases as the vertex angle decreases because by increasing the vertex angle, the tip of the nozzle becomes broader. Therefore, the effective width of the nozzle and capillary force increases, consequently increasing the droplet diameter. In addition, the diameter of a droplet formed in the concave geometry is larger than that of the triangular geometry, and the droplet diameter in the triangular geometry is larger than that of the convex geometry. In fact, according to Fig 8a, for the concave geometry, the tip of the nozzle is blunter than that of the triangular geometry, and subsequently, the tip of the nozzle in triangular geometry is blunter than that of the convex geometry; therefore, the capillary force is maximum in a concave geometry, and minimum in a convex geometry, leading to the droplet formation with a larger diameter in concave geometry compared to the triangular geometry, and larger diameter in the triangular geometry compared to the convex geometry.

In the second case, the effect of symmetry and asymmetry of the hydrophilic part for triangular geometry are compared at different vertex angles (Fig 8c). In asymmetric geometry, the width of the hydrophilic part is less than that of the symmetric triangle with the same vertex angle, reducing the capillary force and the diameter of the droplet compared to the counterpart symmetric triangle. It is also observed that the droplet diameter increases with increasing the vertex angle in the asymmetric geometry as well as the symmetric geometry.

In the third case, droplet formation is studied in trapezoidal geometries with different sides. For this purpose, to accurately compare the trapezoidal geometries, different sections of a triangle with a vertex angle of 10 at different distances from the triangle vertex are considered to create three trapezoids with the top sides of 1.15, 2.3, and 3.5 mm (Fig 8d). It is observed that the smallest droplet diameter has been generated from the triangular geometry, and with increasing the trapezoid side, the width of the hydrophilic part, and subsequently, the capillary force increases, and thus, larger droplets are generated with a lower frequency.

### Theoretical scale analysis

Herein, a scale analysis is carried out to achieve a more profound theoretical understanding of the governing physics of the droplet generation process. Compared to computational fluid dynamics (CFD) [57,58], or other numerical analysis [59–62], scale analysis is more straightforward and can provide a more in-depth understanding of governing forces. First, the effective forces on the process of gravity-driven droplet generation in a sloping wedged-shaped functional surface are discussed, and based on the equilibrium of forces, a scale equation between the droplet diameter and gravitational Bond number is obtained. Secondly, this analysis is expanded to the ferrofluid droplet generation under a magnetic field. Finally, the effect of the geometry of the surface is considered, and an empirical equation is proposed to predict the diameter of the generated droplets.

### Gravity-driven droplet generation

As a hydrophilic-hydrophobic patterned surface is tilted with an angle of α, a component of the gravitational force is generated and exerted on the fluid parallel to the surface direction (x). The magnitude of this force, per unit volume of the fluid, is equal to ρgsin(a), where r is the fluid density (kg m^-3^) and *g* is the earth’s gravitational acceleration (9.81 m s^-2^). This component of the gravitational force pulls the fluid out of the hydrophilic part of the surface toward the hydrophobic part (Fig 2). On the other hand, interfacial forces exerted on the fluid oppose the *g*ravitational force and tend to retract the fluid to the hydrophilic region. Nonetheless, as the driving gravitational force reaches a critical magnitude, it overcomes the opposing interfacial force, and thus the fluid is pulled from the hydrophilic zone, a throat is formed, and eventually, breakup occurs, and a droplet is detached from the fluid pool. Considering the bulk of the fluid in front of the throat as the control mass (ℳ) shown in Fig 2, interfacial forces are exerted on the fluid on the three loci

• ${\mathbb{C}}_{1}$: contact line between the fluid, air, and hydrophobic surface. The force $F_{c,1}$ is exerted on the triple line ${\mathbb{C}}_{1}$, the magnitude of which in the x-direction is calculated by Equation (2) [1], where γ is the liquid-gas surface tension, $\theta_{B}$ is the contact angle between the fluid and the hydrophobic part of the surface, $\vec{n}$ is the unit vector perpendicular to ${\mathbb{C}}_{1}$, and $\vec{i}$ unit vector in the x-direction.

$$F_{c,1}=\int {\mathbb{C}}_{1}\gamma \cos\theta_{B}\vec{n}.\vec{i}dl$$ (2)

**Fig 9:**
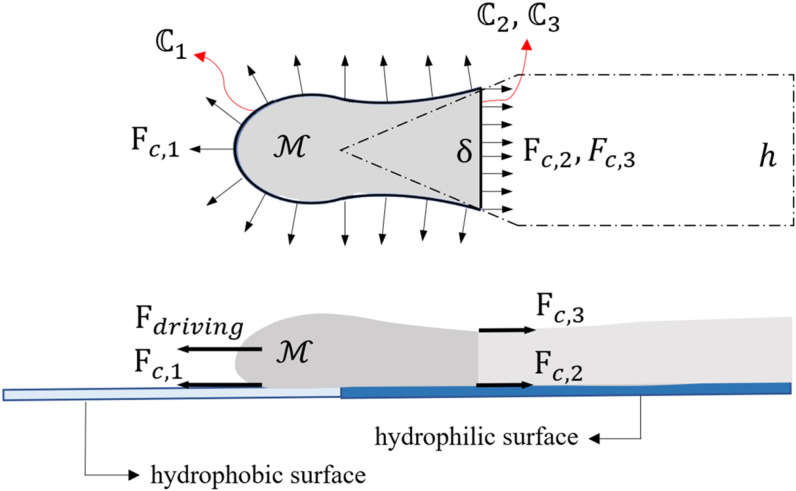
Free diagram of forces exerted on the control mass ℳ.


As the value of $\int {\mathbb{C}}_{1}\vec{n}.\vec{i}dl$ is equal to δ, which is the width of the throat (Fig 9), the magnitude of the force exerted on the triple line can be simplified as in Equation (3):

$$F_{c,1}=\gamma \delta \cos\theta_{B}$$ (3)

- ${\mathbb{C}}_{2}$: Boundary of the control volume on the solid-liquid interface. The magnitude of the force exerted on this line ($F_{c,2}$) is $\gamma_{SL}\delta$, where $\gamma_{SL}$ is the solid-liquid surface tension. It should be noted that as the curve ${\mathbb{C}}_{2}$ is located on the hydrophilic part of the surface, $\gamma_{SL}$ corresponds to the interfacial tension coefficient on the hydrophilic solid surface.
- ${\mathbb{C}}_{3}$: Boundary of the control volume on the liquid-gas interface. The magnitude of the force exerted on this line ($F_{c,3})$ is γ.

For a hydrophilic surface, interfacial energy in the liquid-gas interface is more considerable than that of on solid-liquid interface (${\gamma \gg \gamma }_{SL}$), and thus, the interfacial force exerted on ${\mathbb{C}}_{2}$ could be neglected compared with that of on ${\mathbb{C}}_{3}$. Therefore, the net interfacial force exerted on the control mass M is approximated as Equation (4).


$$F_{c}=F_{c,1}-F_{c,3}=\gamma \delta (1-\cos\theta_{B})$$ (4)

In Equation (4), as the contact angle $\theta_{B}$ is larger than 90 degrees; thus, the net interfacial force scales with γδ. Moreover, assuming that the volume of the fluid at the front of the throat is approximately equal to the volume of the generated droplet, the driving gravitational force scales with $\rho gd^{3}\sin\alpha$, where *d* is the diameter of the generated droplet. Considering a quasi-static process for the droplet generation, the gravitational force applied to the control mass ℳscales with the net interfacial force applied to it, as Equation (5).


$$\rho gd^{3}\sin\alpha \sim \gamma \delta$$ (5)

Also, the width of the throat (δ) is of the order of the width of the hydrophilic part denoted by *h*. Thus, Equation (5) can be rewritten as:


$$\rho gd^{3}\sin\alpha \sim \gamma h$$ (6)

As $Bo=\frac{\rho gh^{2}\sin\alpha }{\gamma }$, a scale correlation between the non-dimensional diameter of the droplet ($\frac{d}{h}$) and gravitational Bond number is derived as follows:


$$\frac{d}{h}\sim Bo^{-\frac{1}{3}}$$ (7)

### Magnetically driven droplet generation

The physics of ferrofluid droplet generation under an external magnetic field is very similar to that of gravity-driven droplet generation, except that the driving force is the magnetic body force rather than the gravitational force. In this case, the magnetic force applied to the unit volume of the ferrofluid can be calculated using Equation (8) [63], where M is the magnetization of the ferrofluid and B is the magnetic flux density.


$$F_{m}=M.\nabla B$$ (8)

Experimentally, as shown previously, the magnetic field of the permanent magnet is almost one-dimensional and horizontal. The magnetic body force can be simplified as Equation (9).


$$F_{m}=M\frac{dB}{dx}$$ (9)

Similar to the previous section, the volume of control mass ℳ scales with $d^{3}$, and the net interfacial force exerted to it scales with γδ, where the width of the throat (δ) is of the same order as the h. In the quasi-equilibrium state for the droplet generation process, these two forces are of the same order as Equation (10).


$$M\frac{dB}{dx}d^{3}\sim \gamma h$$ (10)

Since $B_{m}=\frac{M\frac{dB}{dx}d^{2}}{\gamma }$, the non-dimensional diameter of the generated ferrofluid droplet correlates with the magnetic Bond number as below.


$$\frac{d}{h}\sim B_{m}^{-\frac{1}{3}}$$ (11)

**Fig 10:**
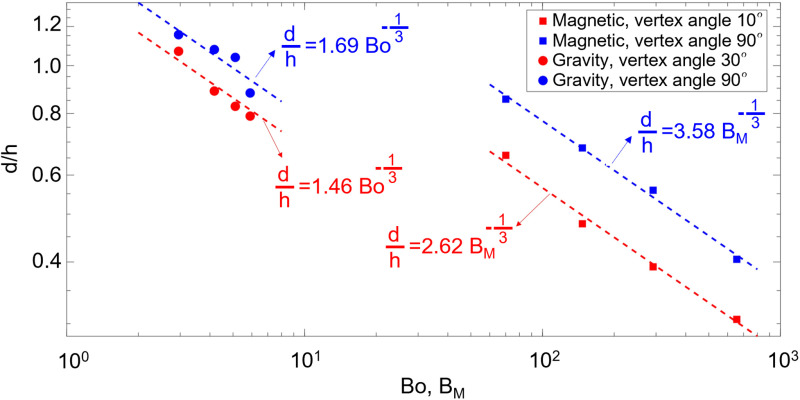
Non-dimensional diameter of droplets versus magnetic and gravitational Bond Number. For these regressions, Pearson’s correlation coefficient (R^2^) is greater than 0.95.

We have examined the validity of the Equations (11) and (7) using experimental data. In Fig 10, the non-dimensional diameter of the droplets is plotted against the magnetic and gravitational Bond numbers. It can be seen that the scale analysis fits well with the experimental data (R^2^ > 0.95), where the diameter of droplets correlates with the gravitational and magnetic Bond numbers with a power of -1/3. As the Bond number increases the droplet diameter decreases logarithmically, that has been previously observed and reported in other nozzles [40,41]. Also, the diameter of droplets increases as the vertex angle of the hydrophilic nozzles increases.

### Effect of geometry

Based on the scale analysis the following correlations are proposed to obtain the diameter of droplets, under the gravitational and magnetic stimuli in Equations (12) and (13), respectively.


$$\frac{d}{h}=\frac{F(G)}{2}Bo^{-\frac{1}{3}}$$ (12)

$$\frac{d}{h}=F(G)B_{m}^{-\frac{1}{3}}$$ (13)

In these Equations, F(G) is the geometry functions, which is an empirical function the value of which is obtained by fitting these equations against the experimental data and is the result of the analysis is shown in Table 2 for different hydrophilic part geometries. According to the experimental data, an interesting observation is that in the case of gravity-driven droplet generation, the coefficient of the gravitational Bond number is half of that of the magnetic Bond number in the case of magnetically driven droplet generation. Also, it should be noted that Equations (12) and (13) can predict the diameter of the droplets with an average and maximum errors of 3.9% and 11.1%, respectively.

**Table 2:** Values of geometry function for some typical hydrophilic surface geometries.

Hydrophilic surface geometry	F(G)
Triangle ($\theta =10^{o})$	2.62
Triangle ($\theta =30^{o})$	3.04
Triangle ($\theta =60^{o})$	3.38
Triangle ($\theta =90^{o})$	3.69
Asymmetric Triangle ($\theta =10^{o}$)	2.27
Asymmetric Triangle ($\theta =30^{o}$)	2.56
Asymmetric Triangle ($\theta =60^{o}$)	3.04
Concave ($\theta =10^{o})$	2.78
Concave ($\theta =30^{o})$	3.11
Concave ($\theta =60^{o})$	3.76
Concave ($\theta =90^{o})$	4.01
Convex ($\theta =10^{o})$	2.53
Convex ($\theta =30^{o})$	3.00
Convex ($\theta =60^{o})$	3.26
Convex ($\theta =90^{o})$	3.54

### Parallel droplet generation

Parallelization of droplet generation is an efficient way to increase the droplet generation rate. Among the common techniques for droplet generation, only step emulsification is capable of parallelization [14]. Here, the capability of the introduced system for parallelization of droplet generation is discussed.

**Fig 11:**
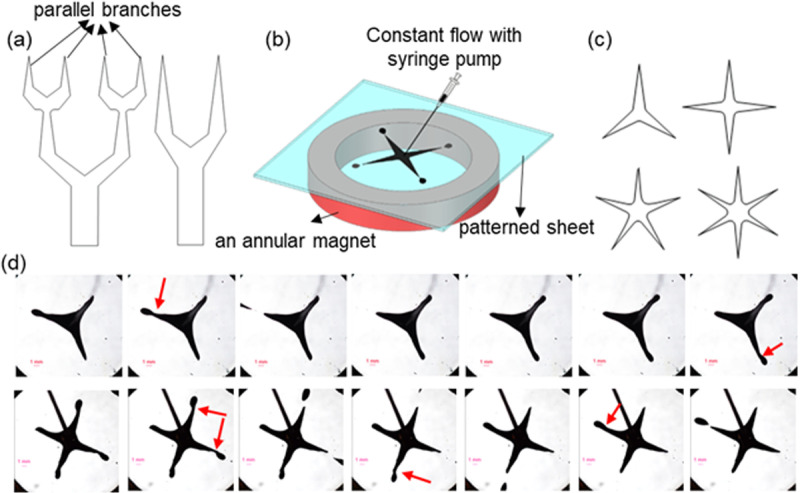
(a) The proposed system for the parallel generation of ferrofluid droplets is inspired by cactus geometry. It involves the formation of two droplets in the right geometry and four droplets in the left geometry. (b) The proposed system for parallel ferrofluid droplet generation uses a star geometry for the hydrophilic part and an annular magnet. (c) The parallel formation of three to six droplets uses stars with three to six vertices. (d) visual comparison of the 3 (first row) and 4 (second row)-vertices star shaped patterns. Red arrows show the instances of droplet pinching off. Time interval between frames showed is 30 ms.

The cactus creates a self-propelled capillary effect to collect water droplets due to the conical geometry of its spines [64]. The two-dimensional geometries presented in Fig 11a, inspired by cactus spines, have been used in reverse mode to generate droplets for the first time in this study. Due to the symmetry in the right geometry, two identical droplets with equal generation frequency (Movie S2) and four droplets with similar diameter and generation frequency are formed simultaneously in the left geometry. The distance between the branches of the right geometry is 7.5 mm, and the distance between the first and fourth branches in the left geometry is 11.5 mm. By changing the flow rate, the magnet distance from the hydrophilic part, and the geometry of the hydrophilic parts, the diameter and generation frequency of the droplets can be adjusted. Also, by patterning an asymmetric branch relative to other branches or creating asymmetric fields, droplets with different diameters can be generated in parallel branches.

Also, using an annular magnet, the possibility of simultaneous and radial generation of ferrofluid droplets is investigated. In Fig 11b, the hydrophilic part is designed in the shape of a star with a radius of 7.5 mm. The glass is mounted on an annular magnet with an inner radius of 25 mm. The fluid moves radially by injecting ferrofluid into the star’s center, and identical droplets are generated with equal frequency from each vertex. Moreover, as depicted in Fig 11c, the parallel generation of three to six droplets is achieved by increasing the number of star vertices from three to six (Movie S3, and Fig 11d). In the experiments, the star and the annular magnet are concentric. With eccentricity, droplets with different sizes and generation frequencies can be created at the star’s vertices.

## Conclusion

This study investigates droplet generation in digital microfluidic systems using a functional-wedged surface with a hydrophilic-hydrophobic design. For the first time, we demonstrated the proposed concept by using water on a sloping surface, showing that smaller droplets with a higher frequency are generated as the tilting angle increases. However, the droplet size remains unchanged as the water flow rate increases while the generation frequency increases. We then examined the formation of ferrofluid droplets on the surface with a hydrophilic-hydrophobic design under an external magnetic field. The droplet sizes and frequency were compared to the state-of-the-art studies and proved higher throughput. The process of ferrofluid droplet formation was observed in five distinct steps: necking, accumulation, vault formation of ferrofluid on the hydrophilic surface at the boundary of hydrophilic-hydrophobic surfaces, overcoming the magnetic force on the interfacial force, forward movement of the ferrofluid at the hydrophobic boundary, droplet formation (breakup), and retraction of the ferrofluid residues to the hydrophilic surface. We begin by exploring the concept through gravity-driven water droplet generation on a sloped surface. Our findings show that smaller droplets are produced at steeper tilting angles, while droplet size remains unaffected by increasing flow rates. Additionally, the frequency of droplet formation decreases by 60% when the tilting angle is reduced from 90° to 30°.

Additionally, taking inspiration from the cactus structure, the system’s ability to parallelize and generate simultaneous droplets was shown.

In addition to the experimental studies, the scale analysis provided a deeper theoretical understanding of the physics governing the droplet generation process. It was found that the non-dimensional droplet diameter is strongly influenced by the gravitational or magnetic Bond number, with both ferrofluid and water droplet formations following a power law of -1/3. An empirical factor was derived by fitting the power law equations to the experimental data, and this factor is tabulated to support future design applications. These equations offer accurate predictions of droplet diameter, with an average error of just 3.9% and a maximum error of 11.1%.

While simulations were not performed in this study, as a future direction of research CFD simulations is recommended to see the effect of multiple parameters that are not feasible investigating experimentally or difficult such as viscosity and density, or where the scale analysis does not provide insight into the problem.

**Nomenclature**
α tilting angle, slope angle
ρ density
g earth gravity
V volume
$V_{d}$ volume of droplet
f frequency
F force
B oBond number
B_m_ magnetic Bond number
F(G) geometry function
d droplet diameter
h width of hydrophilic patterning nozzle
$\theta_{B}$ contact angle at the boundary
$\gamma_{SL}$ solid-liquid surface tension
γ surface tension coefficient
Q flow rate
M magnetization
B magnetic flux density
θ vertex angle
t time
$F_{g}$ gravity force
$F_{c}$ capillary force
$F_{m}$ magnetic force
L magnet distance from the vertex
CV control volume
ℳ control mass
${\mathbb{C}}_{1}$ contact line between the fluid, air, and hydrophobic surface
${\mathbb{C}}_{2}$ boundary of the control volume on the solid-liquid interface
${\mathbb{C}}_{3}$ boundary of the control volume on the liquid-gas interface
$\vec{n}$ unit vector perpendicular to ${\mathbb{C}}_{1}$
δ width of the throat

## Supporting information

Movie S1 Process of Ferrofluid Droplet Generation on a Functional Wedged Surface at θ = 20°, Q = 5 ml h^-‍1^ and L = 10 mm. Video Playback Speed is 0.015x.
(AVI)

Movie S2 Parallel Ferrofluid Droplet Generation on a 2-D Nozzle with 2 Branches. (AVI)

Movie S3 Radial Ferrofluid Droplet Generation on a 2-D Star-Shaped Nozzle with 3 Vertices. (AVI)

Movie S4 Radial Ferrofluid Droplet Generation on a 2-D Star-Shaped Nozzle with 4 Vertices. (AVI)
